# Text Messages to Support Caregivers in a Health Care System: Development and Pilot and National Rollout Evaluation

**DOI:** 10.2196/35318

**Published:** 2022-10-17

**Authors:** Jennifer Lynn Martindale-Adams, Carolyn Davis Clark, Jessica Roxy Martin, Charles Richard Henderson, Linda Olivia Nichols

**Affiliations:** 1 Department of Preventive Medicine University of Tennessee Health Science Center Memphis, TN United States; 2 Veterans Affairs Caregiver Center - Caregiver Support Program Memphis VA Medical Center Department of Veterans Affairs Memphis, TN United States; 3 Office of Connected Care Veterans Health Administration Department of Veterans Affairs San Diego, CA United States

**Keywords:** mobile health, mHealth, self-care, veterans, family caregivers, emotional stress, burden of illness, self-efficacy, mobile phone

## Abstract

**Background:**

Although there are many interventions to support caregivers, SMS text messaging has not been used widely.

**Objective:**

In this paper, we aimed to describe development of the Department of Veterans Affairs (VA) Annie Stress Management SMS text messaging protocol for caregivers of veterans, its pilot test, and subsequent national rollout.

**Methods:**

The stress management protocol was developed with text messages focusing on education, motivation, and stress-alleviating activities based on the Resources for Enhancing All Caregivers Health (REACH) VA caregiver intervention. This protocol was then tested in a pilot study. On the basis of the pilot study results, a national rollout of the protocol was executed and evaluated. Caregivers were referred from VA facilities nationally for the pilot and national rollout. Pilot caregivers were interviewed by telephone; national rollout caregivers were sent a web-based evaluation link at 6 months. For both evaluations, questions were scored on a Likert scale ranging from completely disagree to completely agree. For both the pilot and national rollout, quantitative data were analyzed with frequencies and means; themes were identified from open-ended qualitative responses.

**Results:**

Of the 22 caregivers in the pilot study, 18 (82%) provided follow-up data. On a 5-point scale, they reported text messages had been useful in managing stress (mean score 3.8, SD 1.1), helping them take care of themselves (mean score 3.7, SD 1.3), and making them feel cared for (mean score 4.1, SD 1.7). Texts were easy to read (mean score 4.5, SD 1.2), did not come at awkward times (mean score 2.2, SD 1.4), were not confusing (mean score 1.1, SD 0.2), and did not cause problems in responding (mean score 1.9, 1.1); however, 83% (15/18) of caregivers did not want to request an activity when stressed. Consequently, the national protocol did not require caregivers to respond. In the national rollout, 22.17% (781/3522) of the eligible caregivers answered the web-based survey and reported that the messages had been useful in managing stress (mean score 4.3, SD 0.8), helping them take care of themselves (mean score 4.3, SD 0.8) and loved ones (mean score 4.2, SD 0.8), and making them feel cared for (mean score 4.5, SD 0.8). Almost two-thirds (509/778, 65.4%) of the participants tried all or most of the strategies. A total of 5 themes were identified. The messages were appreciated, helped with self-care, and made them feel less alone, looking on Annie as a friend. The caregivers reported that the messages were on target and came when they were most needed and did not want them to stop. This success has led to four additional caregiver texting protocols: bereavement, dementia behaviors and stress management, (posttraumatic stress disorder) PTSD behaviors, and taking care of you, with 7274 caregivers enrolled as of February 2022.

**Conclusions:**

Caregivers reported the messages made them feel cared for and more confident. SMS text messaging, which is incorporated into clinical settings and health care systems, may represent a low-cost way to provide useful and meaningful support to caregivers.

## Introduction

### Background

Caring for a loved one can substantially impact the emotional and physical well-being of the caregiver. Caregivers report higher levels of psychological distress, depressive symptoms, anxiety, stress, and emotional difficulties than the general population. They also report lower self-ratings of physical health, higher rates of chronic disease, and fewer self-care behaviors [1]. Social support, education, and skills training, for example, coping skills, problem-solving, cognitive reframing, and stress management, have all been shown to be beneficial for caregivers [1]. Multicomponent interventions that can be targeted to caregivers’ specific concerns combining these strategies have generally provided stronger evidence of positive benefit for caregivers [2,3] in the form of reduced depressive symptoms, anxiety, and burden [1,4]. To support caregivers, researchers have tested interventions that vary across format (eg, face-to-face, telephone, inclusion of audio or video, printed materials, and websites), content (single focus or multicomponent), and length (1-48 sessions). Caregiver interventions are generally synchronous and delivered either in-person or by telephone or telehealth [2,5].

Caregivers have been shown to be receptive to technology if the technology is compatible with values and abilities and is tailored toward the caregiver [6]. Reviews of web-based interventions for caregivers of older persons and of persons with dementia show that support by a peer group [7-9], contact with a provider [7,10], assistance with decision-making and problem-solving [7,10], support [7-10], and information [7-10] included as part of a multicomponent intervention have the most positive outcomes. Mobile apps for caregivers also provide them ways to learn [11,12], interact with clinical experts [12], take care of themselves [11,12], and manage the care of their care recipient [12].

There is a need to expand family caregiver support in clinical practice and in diverse populations [3], but clinicians who deliver interventions may not always be available, and organizations may not be willing to invest in web-based interventions or mobile apps. SMS text messaging may be a simple way for health care organizations to address caregiver stress and their need for information. Texting in different formats, including synchronous or asynchronous chats, automated messaging, and push notifications, has been widely embraced as a health care intervention for a variety of conditions and purposes. Different types of text messages have been used, including reminders, information, supportive messages, and self-monitoring procedures. Multiple review articles have examined texting in health care in recent years, concluding that it can be beneficial clinically [13,14]. SMS text messaging improves patient engagement in treatment and outcomes [15,16] and adherence to treatment regimens and medications [17-20].

Despite their success in patient care, texting interventions have not been developed for caregivers [20] other than for parent caregivers of adolescents and children. Integrating SMS text messaging interventions more broadly into health care systems would help support patients, caregivers, and public health [21].

### Text Messaging in the Department of Veterans Affairs

Automated Texting Platform for Veteran Self-Care, part of the Office of Connected Care of the Department of Veterans Affairs (VA), is an SMS capability that can send messages and interpret patient messages following a specified syntax to reply with preprogrammed (bidirectional) responses. Annie cannot decipher or respond without keywords. Veterans and caregivers can access the platform through their mobile phones or smartphones, and veterans also have access to an app. The platform is modeled after the United Kingdom’s National Health Service’s program Flo, after Florence Nightingale, the founder of modern nursing. Flo has been useful in symptom management, for example, in blood pressure control [22] and in early identification and management of complications after colorectal surgery [23]. However, two of the major benefits of Flo are education and feelings of support, control, companionship, and flexibility reported by patients [24]. The program of the VA is known as Annie, named after Lt Annie G Fox, Chief Nurse at Hickam Field, during the attack on Pearl Harbor on December 7, 1941, and the first woman to receive the Purple Heart for combat.

As of February 2022, Annie had 43,229 current users who were registered and had the capability to be on a protocol and 363 different protocols focusing on education and self-care, including disease-specific reminders (eg, diabetes foot care), behavior change encouragement and motivation (eg, exercise and weight loss prompts and breathing and relaxation strategies), medication and symptom monitoring (eg, blood pressure), and treatment adherence reminders and monitoring. Veterans using Annie receive motivational or educational messages that do not require a response or automated prompts to track and monitor their own health. These prompts only allow preprogrammed responses, such as typing in a word to receive further texts or reporting a blood pressure reading. Messages and patients’ data are stored in the Annie system where clinicians can view the texts and readings as needed, but this is not a requirement for clinicians.

Annie, like Flo, not only has clinical benefits but also enhances the satisfaction and empowerment of the patients. Annie reminders have improved adherence to positive airway pressure for patients with traumatic brain injury and sleep apnea with subsequent improvement in sleepiness and cognition [25]. Patients with hepatitis C virus in Annie have shown not only improved adherence to medication regimens but also less distress at failing treatment [26]. Veterans who were receiving chemotherapy used Annie to report symptoms and seek further action if their symptoms warranted it. Patients felt empowered by being able to self-manage their symptoms, particularly when their perceptions of doing well were validated [27]. Like Flo, Annie fosters a sense of connection and confidence in those who use it [28].

The sense of empowerment and companionship made Annie a perfect fit for caregivers. Although Annie had been successful with patients and there was enthusiasm for developing patient protocols, it had not been used for caregivers. The national Caregiver Support Program of the VA provided a receptive environment for providers and caregivers. A small caregiving texting pilot at the University of Tennessee Health Science Center showed that older African American caregivers were receptive to texting. Thus, in 2019, the VA Caregiver Center, part of VA’s Caregiver Support Program, began a collaboration with the VA Office of Connected Care to bring Annie SMS text messaging to caregivers of veterans. This paper describes the development of the Annie Stress Management texting protocol, its evaluation in a pilot study, and the evaluation of the subsequent national rollout into the broader VA system.

### Text Messaging Caregiving Intervention (Pilot)

#### Methods (Pilot)

##### Overview

Caregivers of veterans enrolled in the Caregiver Support Program were eligible for the pilot study as these caregivers all had an electronic health record, a requirement for Annie registration. The pilot study was publicized to the national network of Caregiver Support Program teams of VA located at each facility. Caregiver Support Program staff from each VA facility sent a flyer about the study to each of their caregivers enrolled in the VA Caregiver Support Program. If a caregiver was interested in participating, staff registered the caregiver in Annie and then sent the caregiver’s information to the Caregiver Center Annie coordinator. The Annie coordinator then reached out to the caregiver and completed a short screening interview to ensure that the caregivers could receive text messages and were willing to participate. Once a caregiver agreed to participate, the Annie coordinator mailed consent forms and scheduled a time to go over them over the phone. Caregivers then received messages to help reduce stress for 1 month. After the month was over, the Annie coordinator completed a program evaluation with the caregivers to obtain their feedback on the messages.

##### Ethics Approval

The pilot evaluation was reviewed and approved by the Memphis VA Medical Center institutional review board (IRBNet #1415769). It was conducted from July 11 to November 1, 2019, to evaluate caregivers’ use and satisfaction with the messages and format.

##### Stress Management Caregiver Protocol Development

The Annie Stress Management protocol for caregivers, used in the pilot and the national rollout, is based on the Resources for Enhancing All Caregivers Health (REACH) caregiver intervention used in the Department of VA and the community [4,29,30]. REACH and the text messages are based on the stress health process model [31,32]. As conceptualized by the model, caregivers have challenges and demands placed on them (eg, care of their loved one, lack of help, and their own physical health) and cognitive and emotional responses to these challenges (eg, grief, feeling alone, and viewing events from a negative perspective) [32]. If caregivers do not believe they have resources and the capacity to manage their demands, they experience stress, which can lead to physical and psychological distress and illness [31]. Following the model, the Annie Stress Management text messages focus on knowledge, strategies, and actions that caregivers can take to cope with demands and their responses to them and manage stressors. The goal is to help the caregiver intervene at multiple points in the stress health process (Table 1).

**Table 1:** Stress health process model and Annie message examples.

Model	Annie message examples
Challenges and demands placed on caregivers 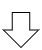	“Getting enough sleep & rest will help you cope and feel better. Tell your doctor you are a caregiver and ask for help.” “Annie here. When you need help or a break from providing care for your loved one it's ok to let friends or family members know.”
Emotional and cognitive response to challenges 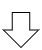	“View stressful situations from a more positive perspective. See a traffic jam as a chance to listen to music or enjoy some alone time.” “Expressing what you’re going through can help even if you can’t change a stressful situation. Talk to trusted friends or go see a therapist.”
Resources and the ability to manage challenges 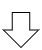	“Keep a stress journal. Make note of when you experience stress to see if there is a pattern. Find ways to remove or lessen those triggers.” “Feel like no one gets you? Try a support group. Ask your Caregiver Support Coordinator https://www.caregiver.va.gov/support/New_CSC_Page.asp”
Perceived stress 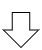	“Anxiety can be managed. Healthy eating, less caffeine & alcohol, relaxing, & time with friends can help. Maybe try meditation or yoga, too.” “Take a deep breath. Gently reach your arms to the side, then reach them out in front of you. Now reach up as high as you can. Repeat.”
Emotional and physical response Illness 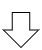	“As a caregiver you're at risk for high blood pressure, heart problems, colds, & flu. Be sure to watch your own health.” “Some caregivers don't have energy for routine tasks. If this sounds like you, seek tips from your doctor or other caregivers.”

##### Caregiver Stress Management Pilot Intervention

The pilot protocol included two types of text messages, those that Annie sent without any caregiver involvement focusing on motivation and education and messages that were requested by the caregiver after a series of prompts by the system and caregiver responses. For the pilot messaging workflow, motivational or educational messages were sent twice a week without any caregiver involvement. The requested messages offered an optional activity to the caregiver. Caregivers were contacted to ask if they were stressed twice a week (on days when a motivational message did not come). If they replied yes, they received a query to ask if they would like to do an activity to help. Caregivers could respond to this activity-requested query to receive content about stress-relieving strategies, breathing, give yourself a break, setting boundaries, and mindfulness. Annie then sent messages to help with stress. For example, for breathing, one stress-relieving strategy was “Annie says to breathe deeply, hold it for 3 seconds. Breathe out slowly. Say a calming word to yourself. Let your jaw, shoulders, and arms go limp. Repeat twice.” Caregivers could contact the text program at any time as many times as they wanted for stress-relieving messages. Each time they texted the appropriate prompt to the system (eg, Activity BREATHING), a message was sent until the bank of 10 to 12 texts for each topic was exhausted and began again. Therefore, caregivers could receive none, one, or several activity messages. Annie provided instructions each time to help the caregivers remember how to request assistance.

##### Evaluation

At the end of a month, caregivers were contacted by telephone to evaluate acceptability of the program’s texts by the coordinator. Questions were focused on technical aspects (eg, easy to read and problems requesting texts) and perceptions of benefit (eg, helped me take better care of myself, made me feel cared for, and made me feel confused). Each question was answered on a scale from 1 (completely disagree) to 5 (completely agree). Data were analyzed using descriptive statistics, including frequencies and means. Open-ended questions asked caregivers what they enjoyed about the messages and why, usefulness of the messages, most helpful messages, use of the messages, and confidence about managing stress after the messages. Caregivers were also asked whether they liked the request prompt messages (responding yes to being stressed and receiving an activity message).

Quotes were examined individually by 2 anthropologist authors, both with prior experience in coding of qualitative data. Each reviewer sorted the descriptions, concepts, and central ideas into potential themes [33] looking for repetitions, similarities, and differences [34]. Topics that occurred repeatedly were linked to verbatim quotes [33]. Themes were discussed and finalized by these authors.

#### Results (Pilot)

Of the 29 eligible caregivers, 22 (76%) caregivers of veterans from 9 facilities returned consent forms and were enrolled in the study. Only one caregiver identified as a male. Baseline demographics for the 22 caregivers showed an average age of 57 years. Most caregivers (18/22, 82%) were White individuals. All the 22 caregivers reported multiple diagnoses for their veterans; the most common reported diagnoses were posttraumatic stress disorder (PTSD; 18), dementia (7), and depression (7). These figures are similar to caregivers of veterans nationally, where 96% are female and 61% are at least aged 50 years, and the top two diagnoses reported for the veterans are mental illness and PTSD [35].

In addition, 82% (18/22) of the caregivers provided follow-up data (Table 2).

Qualitative data yielded 3 themes. Caregivers reported that the messages and activities were helpful and helped them manage stress:

Used breathing exercises—those were great. Take time for myself to calm down and breathe, then rethink the situation.

One caregiver said the following:

I don’t always know what to do when I’m stressed. Reading the messages calms me down. Focuses it elsewhere. Liked the directions to destress. Don’t always think about that.

Caregivers also reported that the texts made them feel like someone cared for them and that they were not alone, which is evident from the following testimony from one caregiver:

Don’t have much family. Nice to know someone cares I’m alive.

Finally, the texts made caregivers take care of themselves and think about their own needs:

I felt that for once it mattered about me. I mattered. I could stop and really think about myself instead of just going through the motions of everyday life.

Comments indicated that caregivers tried the strategies. One caregiver reported about a mindfulness and PTSD distraction activity with an ice cube in the hand:

My favorite activity was the ice one. It made me stop and just watch. Had the Vet do it too when he was upset. Got him to calm down.

The caregivers also felt that the messages were easy to read and convenient, which can be gleaned from the following comment:

I shared with my friend. They were easy to access. Could go back to them as needed.

The caregivers’ responses did not indicate that the messages caused them difficulty.

Three caregivers felt that they had received too many messages. When caregivers were asked about responding to the messages about being stressed and then requesting an activity, 86% (12/14) of the caregivers who answered preferred not to request an activity:

Send out something positive instead of asking for it and ask how your stress level is.

Another caregiver reported that they did not like to think about stress:

Love just getting the messages. Don't like thinking about stress. Asking if I was stressed brought it to the forefront. Don't always realize I’m stressed.

Another caregiver commented the following:

Just give an activity. If you're in a stressful state, you don't know what to do. You're stressed about everything. You don't know what you want/need.

Caregivers were also busy:

Like just getting the messages is easier for me with taking care of my husband and baby.

Another said the following:

Just send an activity. I get busy and don't always realize I'm stressed.

**Table 2:** Annie Stress Management protocol pilot evaluation responses (n=18).

Responses	Completely disagree, n (%)	Disagree, n (%)	Neutral, n (%)	Agree, n (%)	Completely agree, n (%)	Mean (SD)
1. The texts helped me manage my stress.	1 (6)	1 (6)	3 (17)	9 (50)	4 (22)	3.8 (1.1)
2. I feel this program helped me take better care of myself.	2 (11)	1 (6)	4 (22)	5 (28)	6 (33)	3.7 (1.3)
3. I felt like someone cared about my personal well-being when I got the texts.	1 (6)	2 (11)	2 (11)	3 (17)	10 (56)	4.1 (1.3)
4. I would recommend this service to another caregiver.	2 (11)	1 (6)	0	2 (11)	13 (72)	4.0 (1.7)
5. It was easy to read the texts.	0	0	1 (6)	2 (11)	14 (78)	4.5 (1.2)
6. I received the texts at awkward times.	8 (44)	3 (17)	3 (17)	3 (17)	1 (6)	2.2 (1.4)
7. Receiving the texts interfered with my daily life.	15 (83)	3 (17)	0	0	0	1.2 (0.4)
8. I was confused when I received the texts.	17 (94)	1 (6)	0	0	0	1.1 (0.2)
9. I had problems sending in keywords and/or making responses.	7 (39)	8 (44)	1 (6)	1 (6)	1 (6)	1.9 (1.1)

### Text Messaging Caregiving Intervention (National Rollout)

#### Methods (National Rollout)

##### Overview

**Figure 1:**
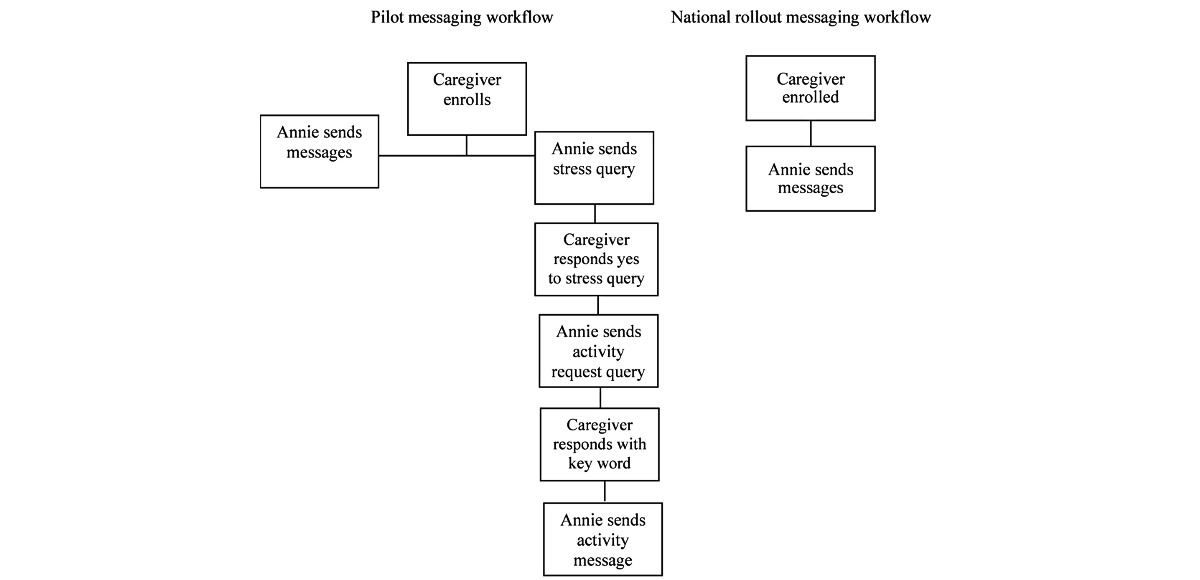
Pilot and national rollout SMS text messaging workflow.

On the basis of the positive response to the messages by caregivers in the pilot study, a national rollout of the Annie caregiver stress management protocol was approved by the VA’s Office of Connected Care for the VA system on October 1, 2019. As the caregivers from the pilot study had expressed a strong preference for being given an activity instead of requesting it, modifications to the protocol were made to have Annie offer messages without the necessity of caregivers replying as shown in Figure 1.

The program was initially publicized through the Caregiver Support Program and the Office of Connected Care of the VA. The eligible candidates were caregivers of veterans enrolled in the VA health care system who had a VA electronic health record or a veteran who was a caregiver. Any VA staff member could register a caregiver from their facility in Annie. Staff at VA facilities determined whether a caregiver was interested in receiving the Annie text messages. If the caregiver was interested, staff then ensured that caregivers had a VA electronic health record, which was a requirement for Annie services, registered the caregiver in Annie, and sent a referral to the Caregiver Center for the caregiver to be entered into the Annie protocol. The Caregiver Center offered training to staff who did not know how to use Annie, and as of February 2022, a total of 592 staff members had been trained.

##### Annie Stress Management Protocol

The Annie caregiver stress management protocol was lengthened to include a year’s worth of messages. On the basis of pilot findings, 3 messages per week, education, motivation or inspiration, and activity were sent with no response required from the caregiver.

In the current version of the protocol, messages are sent 3 times a week on different days and focus on education, motivation or inspiration, and strategies or skills (Table 3). Educational texts provide information about stress and responses to it. Motivational texts, often quotes from famous people, were included on caregiver request and were validating and inspiring. Activities focusing on stress-relieving strategies, breathing, giving yourself a break, taking care of yourself, setting boundaries, and mindfulness are strategies suggested for caregivers to try. General messages were sent to all caregivers in addition to the protocol messages. Messages offering an email link for help or questions and links to the national Caregiver Support Program staff and resources were sent every 3 weeks. Caregivers were registered in Annie by clinical staff at their local VA facility; each caregiver must have an electronic health record. After registration, a referral was sent to the Caregiver Center, which enrolled the caregiver in the requested protocol.

**Table 3:** Annie Stress Management protocol message examples.

Educational (Tuesday 10 AM)	Motivational (Thursday 3 PM)	Activity (Saturday 10 AM)
“Stress is normal in life- it's how we react to threats. Let us help you develop effective ways to manage stress.” [Annie]	“In order to carry a positive action, we must develop a positive vision.” [Dalai Lama]	“Inhale for 4 seconds; hold for 6 seconds; exhale for 4 seconds; hold for 6. Repeat 5 times.” [Annie]
“Holidays can be overwhelming. Avoid taking on too much. Keep things simple. Ask for help. You may need to reduce activities.”	“Annie here. It's important to remember that you are doing a good job taking care of your loved one. Keep it up!”	“Go outside and enjoy the sun and the breeze for a few minutes.” [Annie]
“Annie invites you to listen to a monthly talk about caregiving. https://www.caregiver.va.gov/support-line/presentations.asp”	“The only limit to your impact is your imagination and commitment.” [Anthony Robbins]	“Each day & week do something with your loved one that gives you both pleasure.” [Annie]
“Annie here, with a reminder: Making your family the priority is nothing to feel guilty about.”	“Thank you for taking care of your loved one. Even if no one remembers to tell you, what you are doing is appreciated and makes a difference.”	“Write down 3 things you’re grateful for.” [Annie]

##### Evaluation

At the end of 6 months, caregivers were asked to fill out a brief evaluation survey through a SurveyMonkey (Momentive Inc) link in an Annie message. All data were anonymous. Six questions asked caregivers’ opinions about the texting program using a 5-point Likert scale ranging from completely disagree (score=1) to completely agree (score=5), and data were analyzed with descriptive statistics, including frequencies and means. Caregivers were asked whether the texts had helped manage stress, increase confidence, and take better care of self and loved one. They were also asked whether the texts had made them feel like someone cared about their well-being and whether they would recommend the program.

Two open-ended questions about additional topics and anything caregivers wanted to add were asked. Quotes were examined individually by 2 anthropologist authors, both with prior experience in coding of qualitative data. Each reviewer sorted the descriptions, concepts, and central ideas into potential themes [33] looking for repetitions, similarities, and differences [34]. Topics that occurred repeatedly were linked to verbatim quotes [33]. Themes were discussed and finalized by these authors.

#### Results (National Rollout)

##### Overview

**Figure 2:**
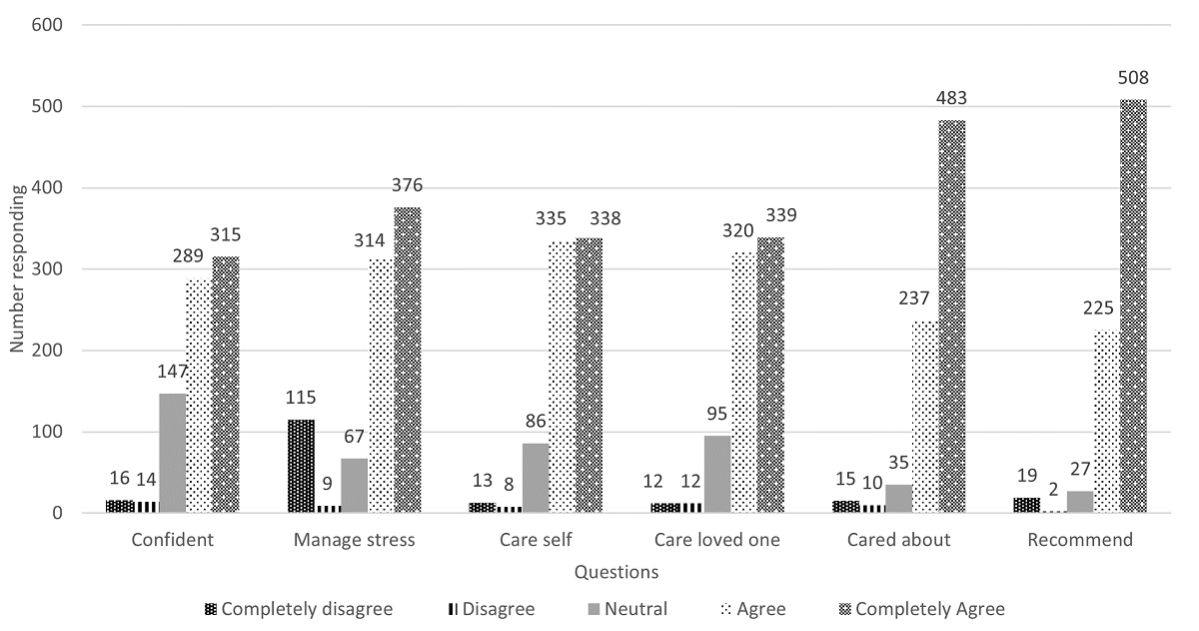
Annie national rollout caregiver stress management text protocol evaluation responses.

In total, 4401 caregivers were enrolled in the stress protocol as of the end of February 2022. All had not been in the protocol for 6 months and were therefore not eligible to receive the evaluation link, with 3522 caregivers eligible. Figure 2 shows the responses from the 781 caregivers who answered the link, a 22.17% (781/3522) response.

Caregivers endorsed 3 messages per week as the right amount (679/778, 87.2%). The mean scores for each question indicated that the caregivers felt that the messages had been useful in increasing their confidence (mean 4.1, SD 0.9), managing their stress (mean 4.3, SD 0.8), and helping them take care of themselves (mean 4.3, SD 0.8) and their loved ones (mean 4.2, SD 0.8). The messages also helped them feel cared for (mean 4.5, SD 0.8). Finally, caregivers would recommend the program to other caregivers (mean 4.5, SD 0.8). In fact, several caregivers reported sharing Annie even on social media. Furthermore, 23.1% (180/778) of the caregivers tried all the strategies, an additional 42% (329/778) tried most strategies, and 26.9% (210/778) tried some strategies. Less than 10% (49/778, 6.3%) tried a few strategies and only 1.3% (10/778) of the caregivers tried none of the strategies.

In their open-ended comments, the caregivers identified 5 general themes, as shown in Table 4. There were only 16 negative comments; most wanted to respond and have Annie answer them (ie, wanted a real person checking on them), and 2 caregivers thought there were too many texts.

**Table 4:** Annie benefit themes and quotes.

Themes	Illustrative quotes
Appreciate texts; bright spot for me; were helpful; shared with others	“I so look forward to Annie. It is a bright spot in my day and helps tremendously.” “The texts have been very helpful, and I know I have needed them more than I knew. And I share many of the messages with my fellow caregiver. I so appreciate having a lifeline.” “These tips and quotes are perfect and truly help my sanity and remind me to breathe.” “I share Annie with my sister who is also a caregiver for her husband and daughter. Annie always seems to address whatever we happen to need help with. We both love receiving texts from our ‘special friend’.”
Right on target; came when I needed them	“Sometimes it was right on target for my emotions and when I least expected it.” “The messages that were given were inspiring and uplifting seemed to come always at the right time. Loved the messages that were personal and uplifting as simple as look at you, you’re doing awesome. Those really helped me out at times and seemed to come at just the right moments. So thank you so much for those!!”
Help with self-care	“The messages were/are a welcomed reminder to ‘self-care.’ Thank you!!!!” “These texts for me were great reminders to take better care of myself and not be too hard on myself. Thank you!” “Annie reminds me when I forget myself sometimes. Thanks Annie!”
Keep them coming; want them to start again	“Just continue sending them...thank you” “VA Annie is just what I need to make my day. Keep them coming!” “Just keep giving me the reminders that I will be able to get us both through this.”
Show me I’m not alone; someone cares	“Just that I am not alone.” “I just like to know that someone cares.” “The messages always come at the times when I need to know I am not alone.”

##### Poststudy Experience

Because caregivers shared the protocol with their veterans, and staff were asking whether veterans could be put on the protocol, a year-long Annie Stress Management protocol for veterans was developed, which is similar in format and types of messages. In February 2022, this protocol enrolled 294 veterans, making it one of the top 10 protocols according to the number of users.

On the basis of the success of the stress management protocol, several new protocols have been developed: bereavement; taking care of you, which focuses on healthy lifestyle and emotions; dementia behaviors, which combines stress management and management of behaviors; and PTSD behaviors, which focuses on coping with behaviors. Similar to the stress management protocol, education and strategies for all these protocols are also taken from the REACH VA behavioral intervention. All protocols except bereavement last for a year. Caregivers can also be enrolled in the VA’s Coronavirus Precautions protocol.

The Annie caregiver protocols had a cumulative total of 7274 caregiver enrollments as of February 2022 with 7062 current users, and each month about 250 caregivers are referred to the Caregiver Center for enrollment in an Annie protocol, showing the feasibility of incorporating SMS text messaging into a large health care system. The Annie caregiver protocols have been extremely successful with more users than many Annie Veteran protocols. For example, in February 2022, among the top 10 Annie protocols based on users, 3 were caregiver protocols. Of the 6386 users in the top 10 protocols, 56.34% (3598) were enrolled in caregiver protocols with 31.99% (2043) of the users enrolled in stress management.

## Discussion

### Principal Findings

In this evaluation of the Annie Stress Management texting pilot and national rollout for caregivers of veterans, caregivers felt that the messages had been useful in increasing their confidence, managing their stress, and helping them take care of themselves and their loved ones. The messages also helped them feel cared for and made them feel less alone, looking on Annie as a friend. The caregivers felt that the messages were right on target and did not want them to stop. These findings are similar to those that veterans have articulated about Annie [27,28].

During the national rollout, caregivers’ requests led to some changes in the stress management protocol, namely, the last week of all the caregiver protocols let the caregiver know that the protocol was ending, thanked the caregiver, and provided the link to request an extension or a new protocol.

Part of the success of the Annie caregiver protocols may be because of their cross-diagnosis applicability and the fact that neither caregivers nor clinicians must respond to the messages. The impact on staff workload does influence the uptake [36]. Research into veteran and staff use of Annie suggests that additional support helps providers adopt, implement, and sustain the program [36,37]. The Caregiver Center has provided this support through coordinator to promote the protocols, help providers with the technology, and train them to use the technology. In addition, because caregivers often rely on others to identify useful technology [6], the relationship between caregiver and VA staff likely facilitates caregiver’s willingness to try SMS text messaging.

One surprising finding was that caregivers in the stress management pilot study reported that they did not want to ask for an activity. In reviews of web-based caregiving interventions, interaction with peers or professionals or interactive support has been shown to be beneficial [7-10]. Despite the lack of 2-way interaction with a clinician or with Annie in the final national rollout protocol, caregivers still found benefit and felt cared for. There may be several reasons for this finding. Caregivers may be too busy for protocols that ask for a response, or they may not want to respond when they are not responding to and receiving answers from a real person. As the text message comes directly to the phone, the messages may feel personal and intimate as a text message from a friend would. The Annie caregiver text programs have positive motivational messages and jokes, two features that are unlike most Annie Veteran facing protocols, which may add to a feeling of being personal. Caregivers did tend to anthropomorphize Annie, thanking her for being there for them.

Our study has some limitations. The lack of negative findings may reflect that people who did not like Annie might have asked for it to be turned off before receiving the 6-month survey link to provide feedback. Another limitation is that the SurveyMonkey data are anonymous; therefore, caregiver characteristics cannot be linked to satisfaction or outcomes. Finally, the 22% response rate is low.

### Conclusions

Although many caregiving interventions are multicomponent and targeted to specific issues of the caregiving dyad [1-4], the stress health process model shows that education, support, and strategies and resources to manage challenges and cope with stress [31,32] are important components of successful interventions. As has been shown with the Flo and Annie texting protocols, these are all part of what users can receive through texting, leading to increased confidence building and empowerment [24,27,28]. For caregivers specifically, research has shown that having concerns acknowledged, perceived attention, and positive regard can all be therapeutic [38], suggesting that any positive contact around caregiving can be beneficial.

This type of SMS text messaging provides a means of reaching many caregivers and is practical for health care systems or clinical practices. Cloud-based messaging systems could be developed with relatively low hosting and per recipient costs. Moreover, texts provide a way to overcome the lack of widespread implementation of interventions into clinical settings, which is a critical barrier to improving outcomes for caregivers and the loved ones they support [1,3,39]. For caregivers, there are similar areas of concern, including self-care, emotional and physical well-being, communication, and stress management. Health care organizations could provide the service to multiple types of caregivers either with or without condition-specific messages, which would be more closely targeted to the dyad’s needs. Text messages can be an adjunct to more traditional therapeutic techniques [40] or used on their own without the caregiver needing to respond to a clinician or to the system. These text messages are perceived as a caring touch from the organization:

To know I’m not alone and that you're thinking of us. Caregiving is very lonely, so a phone call or text saying, hey, how are you today? I'm thinking about you makes a big difference.

Caregiving messaging, incorporated into clinical settings, represents a seamless, low-cost way to provide useful and meaningful support to caregivers, who frequently feel overlooked by the health care system.

## Abbreviations

PTSD: posttraumatic stress disorder
REACH: Resources for Enhancing All Caregivers Health
VA: Veterans Affairs
